# Clinical Outcome of Tuberculous Meningitis with Hydrocephalus — A Retrospective Study

**DOI:** 10.21315/mjms2021.28.5.8

**Published:** 2021-10-26

**Authors:** Davendran Kanesen, Regunath Kandasamy, Albert Sii Hieng Wong, John Tharakan, Chien Joo Lim, Jafri Malin Abdullah

**Affiliations:** 1 Department of Neurosciences, School of Medical Sciences, Universiti Sains Malaysia, Kubang Kerian, Kelantan, Malaysia; 2 Department of Neurosurgery, Hospital Umum Sarawak, Kuching, Sarawak, Malaysia; 3 Clinical Research Centre, Hospital Umum Sarawak, Kuching, Sarawak, Malaysia; 4 Brain and Behaviour Cluster, School of Medical Sciences, Universiti Sains Malaysia, Kubang Kerian, Kelantan, Malaysia

**Keywords:** hydrocephalus, tuberculous meningitis, central nervous system tuberculosis

## Abstract

**Background:**

To study the clinical outcome of tuberculous meningitis with hydrocephalus (TBMH) and the factors contributing to its poor clinical outcome.

**Methods:**

Clinical data of 143 adult patients diagnosed with TBM over a 6-year period in two tertiary hospitals in Malaysia were retrospectively reviewed. Relevant clinical and radiological data were studied. Patients with TBMH were further analysed based on their clinical grade and rendered treatment to identify associated factors and outcome of this subgroup of patients. The functional outcome of patients was assessed at 12 months from treatment.

**Results:**

The mean age of patients was 35.6 (12.4) years old, with a male gender predominance of 67.1%. Forty-four percent had TBMH, of which 42.9% had surgical intervention. In the good modified Vellore grade, 76.5% was managed medically with concurrent anti-tuberculosis treatment (ATT), steroids and osmotic agents. Four patients had surgery early in the disease as they did not respond to medical therapy and reported a good outcome subsequently. Poor outcome (65.2%) was seen in the poor modified Vellore grade despite medical and surgical intervention. Multivariate model multiple Cox regression showed significant results for seizure (adjusted hazard ratio [aHR]: 15.05; 95% CI: 3.73, 60.78), Glasgow coma scale (GCS) (aHR: 0.79; 95% CI: 0.70, 0.89) and cerebrospinal fluid (CSF) cell count (aHR: 1.11; 95% CI: 1.05, 1.17).

**Conclusion:**

Hydrocephalus was seen in 44% of patients in this study. GCS score, seizure and high CSF cell count were factors associated with a poor prognosis in TBM. Patients with TBMH treated medically (TBMHM) had better survival function compared to TBMH patients undergoing surgical intervention (TBMHS) (*P*-value < 0.001). This retrospective study emphasises that TBMH is still a serious illness as 47.6% of the patients had poor outcome despite adequate treatment.

## Introduction

The World Health Organization (WHO) reported approximately 10 million new cases of tuberculosis (TB) in 2018, out of which 1.3 million resulted in death. Approximately 10% of these patients were children and 15% of these patients had presented with extra pulmonary tuberculosis. About 1.7 billion people, accounting to 23% of the world’s population, are estimated to have a latent TB infection and are, thus, at risk of developing active TB disease during their lifetime (1). In Malaysia, TB is an endemic problem and also an important public health issue. The incidence of TB in the general population of Malaysia at present is 79–107/100 000 (1).

Tuberculous meningitis (TBM) is a non-suppurative inflammatory disease of the dura mater and spinal cord meninges caused by tubercle bacillus. It is the most lethal form of tuberculosis. High mortality and neurological disability among survivors are often encountered. Hydrocephalus is one of the most common complications of TBM and is almost always present in patients having disease for 4–6 weeks (2–3). It occurs in approximately 70% patients and is even more common in children (3–5). The varied pattern of clinical features makes the clinical diagnosis of TBM difficult. It is often diagnosed when the brain damage has already occurred (4–7). The emergence of drug-resistant strains has increased in many parts of the world and, therefore, is an emerging therapeutic challenge (1, 8).

When hydrocephalus is the presenting feature, urgent neurosurgical decompression may be required; the underlying TBM should be promptly diagnosed to minimise any delay in the use of specific anti-tuberculous drugs. The clinical implication of hydrocephalus upon presentation in adult patients with TBM is uncertain. The latter could be either of the communicating or the obstructive type, the former being more common. Lamprecht et al. (2), in their study of 217 cases, had managed British Medical Research Council (BMRC) stages II and III TBM having communicating hydrocephalus with medical therapy and reportedly were able to avoid shunt surgery in 70% of these patients. Even in the other 30% who underwent shunt surgery, 41.5% had obstructive hydrocephalus. Although shunting is recommended particularly in obstructive hydrocephalus, surgical relief of hydrocephalus may not alter the neurological status or long-term outcome (9–10). Palur et al. (9), reported that those having grade III and IV of TBM had mortality rates of 51.9% and 100%, respectively, despite cerebrospinal fluid (CSF) diversion procedures. The grade of patients at admission usually determines the management strategy. There are various grading systems for patients of TBM with hydrocephalus (TBMH). One of the commonly used systems is the Vellore grading system proposed by Palur et al. (9). The internal drainage of CSF, in the form of ventriculoperitoneal shunt, has been accepted as the standard of care in patients presenting in good neurological grade (I and II) (9, 11)). There is still no consensus on the treatment protocol for patients of TBMH, presenting in poor neurological grade (III and IV). In general, a trial of external ventricular drain (EVD) is an accepted method of treatment to decide whether a patient will benefit from shunt surgery (12). However, it has been shown that improvement after CSF diversion may take many days or even weeks (11, 13). Prolonged EVD is fraught with the risk of infections.

Thus, a retrospective study at two tertiary teaching hospitals in Malaysia over a 6-year period to study the outcome of TBMH and factors associated with poor clinical outcome was conducted.

## Methods

### Sampling Method

Convenience sampling.

### Study Design and Data

A retrospective cohort study was conducted from two tertiary centres in Malaysia. Data were obtained from patients (aged ≥ 18 years) with a diagnosis of TBM who were admitted and treated in these two centres, viz Hospital Universiti Sains Malaysia, Kubang Kerian, Kelantan and Hospital Umum Sarawak, Kuching, Sarawak, between January 2012 and December 2017 and then analysed retrospectively. The patients’ medical records were reviewed and the following information was collected: demographic characteristics, underlying diseases, clinical features, laboratory data, bacteriology, image studies, use of steroids, anti-tuberculosis treatment (ATT), surgical interventions or drainage, and clinical outcome. Most patients had CSF taken on admission and the following tests were performed: total cell count, glucose, protein, and mycobacterial smears and cultures. Chest radiography and brain computed tomography (CT) scans were performed on all patients upon admission.

### Inclusion Criteria

In our study, all patients are classified as ‘definite’ and ‘probable’ TBM on the basis of standardised clinical case definition that is mentioned in the 2010 article of Marais (14). The criteria used in classification of Marais are as follows:

- Clinical criteria (maximum category score = 6)
- CSF criteria score (maximum category score = 4)
- Cerebral imaging criteria (maximum category score = 6)
- Evidence of tuberculosis elsewhere (maximum category score = 4)

A diagnosis of definite TBM is made when acid-fast bacilli (AFB) are seen, *Mycobacterium tuberculosis* is cultured, or is detected by a reliable molecular method from the CSF in someone with symptoms or signs suggestive of the disease. Probable TBM in cases where imaging is available, a diagnostic score of 12 or above is required. On the other hand, when imaging is not available, a diagnostic score of 10 or above is required. A diagnosis of TBMH is made when there is accompanying radiological evidence of hydrocephalus on the CT brain.

The severity of TBM at the time of admission was assessed using the British Medical Research Council (BMRC) TBM stages (15): i) stage I is defined as a Glasgow coma scale (GCS) of 15 without focal neurological signs; ii) stage II is defined as a GCS of 15 with neurological deficit or a GCS of 11–14 and iii) stage III is defined as a GCS of ≤ 10. Those with TBMH were further graded according to the modified Vellore grade by Mathew et al. (13): i) grade I, had GCS 15 with headache, vomiting, fever ± neck stiffness and no neurological deficit; ii) grade II, represented GCS 15 with neurological deficit; iii) grade III with GCS 9–14 and neurological deficit may or may not be present; and iv) grade IV having GCS 3–8 and neurological deficit may or may not be present.

### Exclusion Criteria

Patients who did not fulfill the diagnostic criteria of TBM, age < 18 years old or an alternative diagnosis to TBM (i.e. cryptococcal meningitis) were excluded from the study.

### Treatment and Outcome

The cases were treated with the classical four-drug ATT (combination of isoniazid [INH], rifampicin [RIF], pyrazinamide [PRZ] and ethambutol [EMB]) for 12 months–18 months. Some cases with prior TB received a five-drug therapy including streptomycin. Dexamethasone was given as an adjunct and tapered off over 4 weeks–6 weeks. Hydrocephalus was treated medically with dehydrating agents, or surgical intervention via EVD, ventriculoperitoneal shunt or a combination of both. The functional outcome of patients was assessed at 12 months post-treatment. These outcomes were based on the Glasgow outcome scale (GOS) as shown below (13):

- Death - dead
- Persistent vegetative state - absent of awareness
- Severe disability - activities of daily living (ADL) - dependent
- Moderate disability - ADL - independent
- Good recovery - full recovery or mild disabilities not affecting daily life

In our study, good recovery and moderate disability were considered a ‘good outcome’ while severe disability, persistent vegetative state or death was reckoned as ‘poor outcome’.

### Sample Size and Study Power

The sample size was calculated based on the objective of this study to compare and analyse outcome of patients with TBMH treated with or without CSF diversion. Based on a dichotomous endpoint and two independent sample groups (TBMH treated medically [TBMHM] and TBMH patients undergoing surgical intervention [TBMHS]), the sample size was calculated as below using Power and Sample Size Programme (Power and Sample Size Calculations version 3.0, Copyright^©^ 1997–2009 by William D Dupont and Walton D Plummer):

- Alpha: 0.05
- Beta: 0.2
- Power: 0.8
- Incidence in Group 1: 70% (Good outcome in TBMHM)
- Incidence in Group 2: 30% (Good outcome in TBMHS)

Data published by Lamprecht et al. (2) on the management of TBMH was used as a reference to calculate the sample size. A minimum of 23 patients in each arm is required to achieve the above study parameters. Thereby, the calculated sample size is 46 patients. If a dropout rate of 15% is considered into the sample, a total sample of 54 patients is required.

### Statistical Analysis

Statistical analysis was performed with SPSS version 22.0 (SPSS, Inc., Chicago, IL, USA). Data were first explored and screened. Continuous variables were presented in mean and standard deviation or median and interquartile range. Categorical variables were expressed as frequency and percentage. Univariate Cox Regression analysis was used to explore the associated factors for poor GOS outcome followed by Multivariate Cox Regression. Kaplan Meier survival curves were used to compare TBMHM and TBMHS. A *P*-value of < 0.05 was regarded as significant.

## Results

### Clinical Descriptive Data

A total of 164 patients’ clinical records were reviewed from both centres; however, 21 (12.8%) records were excluded from the study as they did not fit the inclusion criteria or were incomplete data. From the studied cohort, 143 patients had a mean (SD) age of 35.6 (12.4) years old. Majority of them were male (67.1%). Only 10.5% of patients were diagnosed with definite TBM and the rest were probable TBM based on Marais criteria. The most common presenting symptoms in TBM according to order were fever (86.7%), neck stiffness (63.6%), constitutional symptom (33.6%), altered consciousness (30.1%), raised intracranial pressure symptoms (28.7%), hemiplegia (23.8%), cranial nerve palsies (10.5%) and seizure (9.1%). Mantoux test results were positive in 58.7% of patients. Abnormal chest X-ray findings suggestive of TB were seen in 51% of patients. Positive CT brain findings were cerebral oedema (56.6%), hydrocephalus (44.1%), basal enhancement (32.2%), tuberculoma (14.7%), and infarcts (11.9%). A negative CT finding was seen in 9.1% of patients. All patients received ATT and 85.3% had steroids as an adjunct. Forty-four percent had TBMH, of which 42.9% had surgical intervention for the management of hydrocephalus. Table 1 summarises the clinical and laboratory findings in our patients.

Descriptive analysis was used to study the treatment rendered in the good and poor modified Vellore grade, as the numbers were small in this subgroup of patients. All patient in the good grade had a better outcome, of which only four had CSF diversion procedures and the remaining was managed medically (Table 2). In the poor grade, only two patients benefitted from surgery and the other 21 patients had poor outcome despite CSF diversion procedures (Table 3).

### Risk Factors for Poor Outcome in Tuberculous meningitis

**Figure 1:**
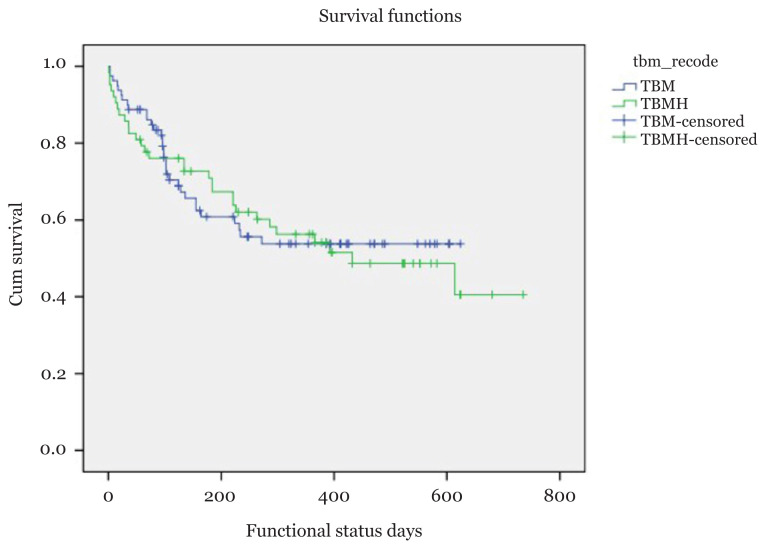
Kaplan-Meier survival curves showing the median survival time for patients with TBM and TBMH

**Figure 2:**
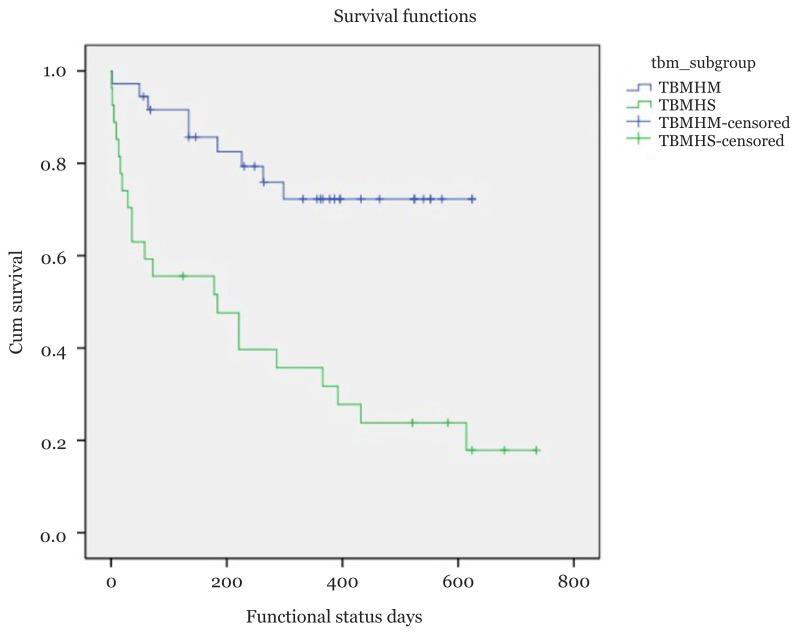
Kaplan-Meier survival curves showing the median survival time for patient with TBMHM and TBMHS

A total of 17 (11.9%) patients died, 17 (11.9%) remained in persistent vegetative state, 28 (19.6%) had severe disability, 34 (23.8%) manifested moderate disability and 47 (32.9%) showed good recovery. These functional outcomes were further grouped into good outcome (moderate disability and good recovery) and poor outcome (death, persistent vegetative state and severe disability). Event was defined as poor outcome and censored for good outcome. Figure 1 shows the median survival time for patients with TBMH was 432 days. The median survival time for TBM without hydrocephalus was not calculated as the smallest survival function did not reach 0.5 or below. Figure 2 shows the Kaplan-Meier survival curve for patients with TBMHM and TBMHS. Patients with TBMHM had better survival compared to those with TBMHS (*P*-value < 0.001). Simple Cox regression was used to explore the significant variables to be included in multivariate model. Multivariate model multiple Cox regression only showed significant results for seizure (adjusted hazard ratio [aHR]: 15.05; 95% CI: 3.73, 60.78), GCS (aHR: 0.79; 95% CI: 0.70, 0.89) and CSF cell count (aHR: 1.11; 95% CI: 1.05, 1.17). Table 4 summarises all the risk factors for poor outcome in TBMH following simple and multiple Cox regression.

## Discussion

Before *Mycobacterium tuberculosis* was identified by Robert Koch in 1882, TBM was clinically described by Robert Whytt in 1762 for the first time in children with acute hydrocephalus (7). Until the discovery of anti-TB drugs in the second half of the 20th century, TBM was considered a fatal disease. However, its mortality can still reach 60%, particularly in developing countries. Sequelae can be seen in 25% of survivors despite five major and numerous minor drug options available (16–17). Advanced disease stage and delay in therapy are considered poor prognostic factors; so, early diagnosis and treatment are important.

The definitive bacteriological diagnosis of TBM depends on the demonstration of *Mycobacterium tuberculosis* by smear or culture in CSF, meninges or brain tissue. Confirmatory CSF culture isolation and polymerase chain reaction (PCR) for TBM are known to have low yield and sensitivity; this by itself presents another challenge to an already constrained setting (18–19). Positive culture has been found in 12%–74% of patients (8, 20–22). In our study, the rate of bacteriological diagnosis was lower (10.5%) than most other large studies. This was via direct smear for AFB, as cost was a limiting factor for TB-PCR or GeneXpert then. Mantoux test results were positive in 58.7% of patients; however, this result alone is not specific for the diagnosis of TBM, as it has been reported in various literatures ranging from 39%–85% in TBM confirmed patients (19, 21). With these confounding factors in place, classifying patients according to the Marais was a challenge in our study. Majority of them were grouped into the ‘probable’ and ‘possible’ TBM categories as the ‘definitive’ TBM yield was seen in only 10.5% of our study cohort. The rest were diagnosed by the treating physicians based on the clinical and radiological data available. Abnormal chest X-ray findings were seen in 51% of our patients, which was within the reported incidence of its occurrence (44%–71%) (19, 21).

During hospital admission, 45.4% of the cases were TBM stage II and 22.4% were TBM stage III. In our study, we had similar results for patients in stage III (22.4%) upon admission, compared to series from Turkey (22.4%) and Northern Taiwan (25.9%) (7–8). This reduction in number compared to previously reported studies could be largely due to the advancement in rural and district health particularly in our country. The BMRC staging of TBM depends on the neurological signs and state of consciousness on admission. Previous studies indicate a correlation between the severity of TBM and poor outcome (19, 21, 23) and this was also seen in our study.

There have been many studies on poor prognostic factors in TBMH, some of which were advanced age, low GCS on admission, concomitant TB at other sites and BMRC stage III on admission (8, 28, 30). Table 4 summarises all the risk factors for poor prognosis in TBMH following simple and multiple Cox regression. Seizure, admission GCS and CSF cell counts were significant factors as evident following multiple Cox regression. Those who presented with seizures has 15 times higher risk of developing poor outcome compared to those without seizure (*P* < 0.001; 95% CI: 3.73, 60.78). Seizure is a known finding in patients with TBM, with reported increased incidence of death up to four times and severe neurological deficit (29). The ongoing brain inflammation, diffuse neuronal injury and reactive gliosis has been attributed to this. In the early phase, seizure is commonly due to meningeal irritation and cerebral oedema. In the later phase, they are usually due to infarction, hydrocephalus, tuberculoma and paradoxical response. This may result in recurrent uncontrollable seizures or progress into status epilepticus which is associated with poor prognosis. A unit increase in GCS score in patient will decrease the risk of developing poor outcome by 21% (*P* < 0.001; 95% CI: 0.70, 0.89), whereas a unit increase in the CSF cell count will increase the risk of developing poor outcome by 11% (*P* < 0.001; 95% CI: 1.05, 1.17). Gu et al. (30) alongside with many other authors have reported poor GCS and hydrocephalus at presentation as an independent factor for poor prognosis (31). This clinical presentation is due to the severe ongoing brain inflammation, again relating to seizure electrolyte imbalances and hydrocephalus. Brainstem strokes are another common cause of low GCS particularly in patients with TBM, due to cerebral vasculitis. Aggressive and prompt management of disease particularly in the early phase with ATT and steroids as per clinical guidelines is of utmost importance here, to prevent the sequalae of TBM.

The incidence of TBMH has been reported up to 70% in recent literatures (3–5, 24–26). In our study, 44.1% of patients had hydrocephalus on neuroimaging. TBMH could be either the communicating or the obstructive type, with the former being more common (4). In either stage of the disease, the thick gelatinous exudates block the subarachnoid spaces in the base of the brain (notably the interpeduncular and ambient cisterns), leading to communicating hydrocephalus (27). TBMH has been reported in various literatures to have an unfavourable impact on the prognosis (24, 28). In our study, 65.2% of patients in the poor modified Vellore grade had poor outcome, whereas all patients in the good modified Vellore grade had a good outcome. Further analysis of patients in the poor modified Vellore grade showed that only two patients had good outcome following surgery, and the remaining 30 patients despite CSF diversion and ATT reported a poor outcome (Table 3). In the good modified Vellore grade, 76.5% (*n* = 13) was managed medically with a combination of ATT, steroids and osmotic agents. Four patients had surgery early in the disease as they did not respond to medical therapy and reported a good outcome subsequently (Table 2). Figure 2 showed that patients with TBMHM (medical management) had better survival as compared to TBMHS (surgical management). This was partly due to the poor pre-operative grades of the patients undergoing CSF diversion procedures, which was 85.2% (*n* = 23) (Table 3). Rajshekhar (31) in his review article, reported a high mortality in excess of 80% in those with poor grade. The authors here opine that, whenever hydrocephalus is a presenting feature in TBM, rapid management in the form of CSF diversion procedures should be offered particularly in those with good modified Vellore grades to prevent this group of patients to further deteriorate. The marginal difference between those with modified Vellore grades II and III, makes it imperative to monitor these patients closely preferably in an intensive care unit to ensure that if any symptoms suggestive of raised intracranial pressure from hydrocephalus can be promptly identified and treated. A lumbar puncture to measure the intracranial pressure particularly in those borderline cases would be a good alternative to aid in monitoring the intracranial pressure for a definite treatment. Some of which as seen in our study, may just respond to medical therapy without any need of surgical intervention. As mentioned above, the outcome of those with poor modified Vellore grades (III and IV) is generally poor, a trial of EVD is still practiced in our centres for 48 h to look for any clinical improvement as in some cases, the hydrocephalus may be a leading factor resulting in a lower grade. Ideally, we would like to have a magnetic resonance imaging (MRI) brain at this particular stage to look for brainstem vasculitic strokes which may also be the causative factor for a poor conscious state, however due to logistic purposes this was not always possible. Following CSF drainage, if a there is a clinical improvement then a permanent form of CSF diversion in the form of a shunt is placed. In the event if no clinical response is achieved, a multi-disciplinary meeting is held along with the family for direction of care, mostly being conservative and supportive in nature.

## Conclusion

Patients receiving medical therapy had better survival than those requiring CSF diversion procedures for TBMH. In our study cohort, majority of the patients were males. Fever and neck stiffness were the most common presenting symptom. Hydrocephalus was seen in 44% in this study. GCS score, seizure and high CSF cell count were factors associated with a poor prognosis in TBMH. In the subgroup descriptive analysis (Table 2), the good modified Vellore grade had good outcomes regardless of the method of treatment. However, further study to determine its significance needs to be conducted prospectively. Patients with TBMHM had better survival function compared to those with TBMHS. Based on our limited numbers, this was partly due to the poor pre-operative grades of these patients. Prompt and early management of hydrocephalus in this group of patients may alter the clinical outcome. Finally, this retrospective study emphasises that TBMH is still a serious illness, as 47.6% of these patients had poor outcome despite adequate treatment.

## Study Limitations

There were a few noticeable limitations in this study, firstly being the management and timeliness of the referral to the neurosurgical unit for the management of hydrocephalus. Not all patients with TBMH was referred to the neurosurgical unit upon diagnosis. Majority of them were referred in the later stages of the disease or following neurological deterioration due to hydrocephalus. This could be the reason why the patients in TBMHS had a worse off outcome compared to TBMHM. Secondly, not all patients had an MRI done during hospitalisation as cost was a limiting factor. This is an important modality to rule out other causes of reduced consciousness such as brainstem infarcts in a patient with confounding TBMH. Due to the retrospective nature of this study, the interrater variability was not calculated. The diagnosis of TBM was based on the clinico-radiological diagnosis by the treating physician and radiologist. As the treating physician/radiologist are not constant, the author does agree that there would be a certain degree of interrater variability in the diagnosis and management of TBM or TBMH in this study. Lastly, being a retrospective study, the advantages of a prospective randomised study for direct comparison was not possible. Hence, a future prospective study comparing this management dilemma will be of great significance.

## Figures and Tables

**Table 1:** Clinical, surgical and laboratory characteristics in patients with TBM (*n* = 143)

	Mean (SD)	Median (IQR)	Frequency (%)
**Clinical data**			
Duration of symptoms*			
Acute (< 2 weeks)			16 (11.2)
Subacute (2–8 weeks)			92 (64.3)
Chronic (> 8 weeks)			35 (24.5)
TB history			
TB contact			32 (22.4)
Co-existing TB			14 (9.8)
Not available/unknown			97 (67.8)
GCS	12.43 (3.20)	14 (3)	
TBM grade†			
Stage I			46 (32.2)
Stage II			65 (45.4)
Stage III			32 (22.4)
Modified Vellore grading‡			
Grade I			7 (11.1)
Grade II			10 (15.9)
Grade III			36 (57.1)
Grade IV			10 (15.9)
Pupils			
Normal			118 (82.5)
Unequal			13 (9.1)
Dilated			12 (8.4)
Fundus			
Normal			40 (28.0)
Papilledema			20 (14.0)
Not available			83 (58.0)
Functional status (GOS)**			
Death			17 (11.9)
Persistent vegetative state			17 (11.9)
Severe disability			28 (19.5)
Moderate disability			34 (23.8)
Good recovery			47 (32.9)
**Surgical intervention for TBMH**			
EVD			8 (29.6)
Shunt			3 (11.2)
Both			16 (59.2)
**Laboratory data**			
Peripheral WBC (×10^3^/μL)	8.98 (3.70)		
Serum Na^+^ (mmol/L)	128.79 (6.51)		
ESR −mm/h	46.97 (25.74)		
HIV			
Positive			21 (14.7)
Negative			97 (67.8)
Not available			25 (17.5)
CSF for AFB			
Positive			15 (10.5)
Negative			121 (84.6)
Not available			7 (4.9)
CSF cell count (cells/μL)	15.27 (8.61)		
CSF protein (> 0.5g/L)	1.66 (1.06)		
CSF glucose (mmol/L)	2.69 (0.84)		
Random serum glucose (mmol/L)		5.70 (1.10)	

Notes:

* symptoms related to TBM;

† based on BMRC TBM grade on admission;

‡ grading for TBMH;

** functional status at 12 months; WBC = white blood cell; ESR = erythrocyte sedimentation rate; HIV = human immunodeficiency virus

**Table 2:** Descriptive data of TBMH treatment in the good modified Vellore grades (I and II) and outcome (*n* = 17)

	Good modified Vellore grade (I and II)				

	EVD	Shunt†	EVD + shunt†	Medical*	Total
Good outcome	0	2	2	13	17
Poor outcome	0	0	0	0	0

Total	0	2	2	13	17

Notes:

† ventriculoperitoneal shunt;

* osmotic agents

**Table 3:** Descriptive data of TBMH treatment in the poor modified Vellore grade (Grades III and IV) and outcome (*n* = 46)

	Poor modified Vellore grade (III and IV)				

	EVD	Shunt†	EVD + Shunt†	Medical*	Total
Good outcome	0	0	2	14	16
Poor outcome	8	1	12	9	30

Total	8	1	14	23	46

Notes:

† ventriculoperitoneal shunt;

* osmotic agents

**Table 4:** Risk factors for poor outcome in TBMH (*n* = 63*)*

	Simple Cox regression			Multiple Cox regression		
	
	b	Crude HR (95% CI)	*P*-value	b	aHR (95% CI)	*P*-value
Fever						
No	0.00	1.00	–			
Yes	−3.01	0.05 (0.01, 0.48)	0.009			
Neck stiffness						
No	0.00	1.00	–			
Yes	1.52	4.59 (1.39, 15.17	0.013			
Altered consciousness						
No	0.00	1.00	–			
Yes	1.78	5.90 (2.83, 12.31)	< 0.001			
Seizure						
No	0.00	1.00	–	0.00	1.00	–
Yes	3.08	21.64 (6.35, 73.72)	< 0.001	2.71	15.05 (3.73, 60.78)	< 0.001
Raised ICP symptoms						
No	0.00	1.00	–			
Yes	1.14	3.12 (1.33, 7.30)	0.009			
TB history						
TB contact	0.00	1.00	–			
Co-existing TB	2.30	10.00 (2.00, 49.95)	0.005			
Not available	1.24	3.47 (0.81, 14.76)	0.093			
GCS	−0.33	0.71 (0.65, 0.78)	< 0.001	−0.24	0.79 (0.70, 0.89)	< 0.001
Modified Vellore grade						
Good grade*	0.00	1.00	0.023			
Poor grade**	3.68	39.58 (1.66, 944.77)				
Pupils						
Normal	0.00	1.00	–			
Unequal	2.15	8.59 (3.36, 21.96)	< 0.001			
Dilated	2.03	7.60 (3.16, 18.26)	< 0.001			
Serum Na^+^(mmol/L)	−1.95	0.82 (0.77, 0.88)	< 0.001			
HIV status						
Positive	0.00	1.00	–			
Negative	−1.34	0.26 (0.12, 0.58)	0.001			
Not available	−2.22	0.11 (0.02, 0.50)	0.004			
CT brain - Infarcts						
No	0.00	1.00	–			
Yes	2.28	9.78 (4.31, 22.20)	< 0.001			
CSF for AFB						
Negative	0.00	1.00	–			
Positive	2.07	7.96 (3.29, 19.27)	< 0.001			
CSF cell count (cells/μL)	0.12	1.13 (1.09, 1.18)	< 0.001	0.10	1.11 (1.05, 1.17)	< 0.001
CSF protein (> 0.5 g/L)	0.92	2.51 (1.85, 3.40)	< 0.001			
Glucose CSF	−3.19	0.04 (0.01, 0.13)	< 0.001			

Notes: Forward LR Cox proportional hazards regression model applied; log-minus = log plot, hazard function plot and partial residuals were applied to check the model assumption and found fulfilled; ICP = intracranial pressure; HIV = human immunodeficiency virus;

* modified Vellore grade I and II;

** modified Vellore grade III and IV
